# A Case of Cancrum Oris in Cook County Hospital

**Published:** 1872-05-01

**Authors:** E. Fletcher Ingals

**Affiliations:** House Physician


### A CASE OF CANCRUM ORIS IN COOK COUNTY HOSPITAL.

REPORTED BY E. FLETCHER INGALS, M.D.,

(*House Physician*).

O— G—; æt. 8. Admitted to the hospital September 14th. His previous history, kindly furnished me by Dr. L. W. Case, is in substance as follows:

The patient was first taken sick August 19th, in the same house where his mother and two sisters were suffering from typhoid fever. In the inception of the disease, the symptoms in this case nearly resembled those which had characterized the beginning of the fever in the mother and sisters, though they inclined more to those of remittent. During the first week, constipation existed; but subsequently diarrhœa was present most of the time, with four or five stools daily. These symptoms were treated with dilute nitric and hydrochloric acids, with an occasional opiate to control the action of the bowels. As much milk and beef tea as the patient could take, were ordered; but as most of the family were sick, the lad seems to have been neglected, and it is probable that directions with regard to medicine and diet were not well followed.

September 1st. Pulse 128; sudamina on chest and abdomen; no appetite; no stools; puffiness under left eye; urine normal; no pain.

September 2d. Patient appeared better.

September 3d. The swelling had increased, and the pulse ranged at 140; patient weak. Quinine was prescribed, and the administration of beef tea and milk insisted upon.

The swelling gradually increased. On the evening of the 6th, the Doctor noticed that the breath was extremely offensive; but the poor light, and the swollen condition of the cheek, prevented him from determining the seat of the difficulty. The following morning he discovered a dirty-looking spot, apparently a slough, on the inside of the left cheek, opposite the upper molars. Carbolized water was used as a disinfectant. On the following day, eight days after the first puffiness was observed, a small black spot appeared on the cheek externally; and twelve hours later, the cheek was found to be perforated. By this time, several teeth had fallen out in consequence of the action of the disease upon the alveolar processes. Portions of the slough were removed, and the carbolic acid dressing, with poultices, again applied. From this time sloughing continued; and five days later, at the urgent request of the parents, who feared that the disease was contagious, the lad was sent to hospital.

On admission, September 14th, the left cheek was entirely gone. The slough which remained adherent involved the ala of the nose, and the left half of both; it extended downwards to near the lower border of the inferior maxilla, backwards to the ear, and upwards to the orbit. The left eye was closed, and the swelling had extended to the left temple.

In this condition, articulation was of course very imperfect, though the nurse soon understood the boy's almost constant cry for water. When fluid was given, a portion was swallowed, but a large part escaped through the side of the face. The excessive fetor, together with all this, made an object inconceivably pitiable and hideous.

In response to the patient's cry for water, milk was given to allay thirst and furnish nourishment; carbolized dressings were applied; no pain was complained of.

September 15th, A.M. Swelling had increased upon the temple, and began to appear on the scalp; no pain. The carbolized dressings were continued, and wine, beef tea, and milk administered.

P. M.—Swelling of temple and scalp increased; temple emphysematous; all of the left half of scalp swollen. Patient objected to milk. Wine and beef tea were continued.

September 16th. The nurse reports that during the night the little patient seemed perfectly rational, and called often for his mother. At half past six in the morning, the patient became unconscious, and continued in this state until 7 o'clock, when he died.

